# Bioactives from Crude Rice Bran Oils Extracted Using Green Technology

**DOI:** 10.3390/molecules28062457

**Published:** 2023-03-07

**Authors:** Donporn Wongwaiwech, Sudthida Kamchonemenukool, Chi-Tang Ho, Shiming Li, Nutthaporn Majai, Tepsuda Rungrat, Kawee Sujipuli, Min-Hsiung Pan, Monthana Weerawatanakorn

**Affiliations:** 1 Department of Agro-Industry, Rajamangala University of Technology Lanna Tak, 41/1 Moo 7, Mai Ngam, Mueang, Tak 63000, Thailand; 2 Department of Agro-Industry, Naresuan University, 99 Moo 9, Tha Pho, Mueang, Phitsanulok 65000, Thailand; 3 Department of Food Science, Rutgers University, 65 Dudley Road, New Brunswick, NJ 08901, USA; 4 Department of Food Science, College of Life Sciences, Huanggang Normal University, Huanggang 438000, China; 5 Department of Agricultural Science, Faculty of Agriculture, Natural Resources and Environment, Naresuan University, 99 Moo 9, Tha Pho, Mueang, Phitsanulok 65000, Thailand; 6 Institute of Food Science and Technology, National Taiwan University, No.1, Section 4, Roosevelt Road, Taipei 10617, Taiwan

**Keywords:** oryzanol, phytosterol, policosanol, supercritical extraction, subcritical extraction

## Abstract

Crude rice bran oils from different rice cultivars and extraction methods bear different contents of nutraceuticals. The health benefits of lowering cholesterol activity of rice bran oil being confirmed by many reports are partly attributed to non-nutrient nutraceuticals, especially γ-oryzanol, phytosterols, and policosanols. As the world has been facing the global warming crisis, green extraction technology is gaining attention from many sectors. The current study aims to compare the nutraceutical composition with respect to γ-oryzanol, phytosterol, and policosanol content as well as the antioxidant properties of crude rice bran oils extracted from white and red rice bran using three green technologies, comparing with conventional hexane extraction. The data show that the traditional solvent extraction gave the highest oil yield percentage (26%), but it was not significantly different from subcritical liquefied dimethyl ether extraction (24.6%). Subcritical liquefied dimethyl ether extraction gave higher oil yield than supercritical CO_2_ extraction (15.5–16.2%). The crude rice bran oil extracted using subcritical liquefied dimethyl ether extraction produced the highest total phenolic contents and antioxidant activities. The highest γ-oryzanol content of the crude rice bran oil was found in oil extracted by conventional cold press (1370.43 mg/100 g). The γ-oryzanol content of the oil obtained via subcritical liquefied dimethyl ether extraction was high (1213.64 mg/100 g) compared with supercritical CO_2_ extraction. The red rice bran yielded the crude rice bran oil with the highest total phytosterol content compared with the white bran, and the oil from red rice bran extracted with subcritical liquefied dimethyl ether generated the highest total phytosterol content (1784.17 mg/100 g). The highest policosanol content (274.40 mg/100 g) was also found in oil obtained via subcritical liquefied dimethyl ether extraction.

## 1. Introduction

Rice bran oil, a high-value-added edible oil, is known for being a rich source of nutrients, such as unsaturated fatty acids, tocopherols, and tocotrienols [1], and non-nutrient nutraceuticals, in particular γ-oryzanol, phytosterols, and policosanols, which exhibit various health benefits, in particular a cholesterol-lowering effect, reducing cardiovascular disease [2,3,4,5]. Many reports on the health benefits of rice bran oil consumption have been established, such as antioxidant property, anti-inflammatory activity, anti-cancer, lowering cholesterol absorption etc. [6,7,8,9,10], and among them, the lowering blood cholesterol property is the most outstanding one. The cholesterol-lowering activity of rice bran oil is proposed by different mechanisms, including decreasing cholesterol absorption, inhibiting the enzyme involved in cholesterol synthesis [11,12,13], leading to prevention of cardiovascular diseases and some cancers [14,15,16,17]. Apart from the balanced fatty acid composition containing a 1.8:1.4:1 ratio of monounsaturated: polyunsaturated: saturated fatty acids [18], the presence of cholesterol-lowering nutraceuticals in rice bran oil, especially γ-oryzanol, phytosterols, and policosanol, clearly explains such benefits.

The most popular conventional extraction method of rice bran oil for cooking oil production is solvent extraction techniques (hot extraction), and hexane is the most used solvent worldwide. For functional rice bran oil as dietary supplement, the cold-pressed extraction method as a conventional green technique is widely used [18,19,20]. Organic solvent extraction gave a high yield of rice bran oil [21,22], but this requires a high temperature, leading to bioactive degradation and energy-intensive steps, including evaporation for solvent removal. Moreover, there is increased consumer interest in organic food and green processed food produced in a safe, economical, and environmentally friendly way. In this context, supercritical fluid extraction (SFE) and subcritical extraction (SUBE) have emerged as eco-friendly processes to overcome the toxicity drawbacks associated with conventional solvent extraction. Both SFE and SUBE, as green techniques, diminish the damage caused to the environment and offer high-quality oil products. Carbon dioxide is the most common solvent employed for the SFE extraction process, whereas propane, butane, dimethyl ether (DME), and water are used as the solvent for subcritical extraction [23,24,25,26,27,28,29].

Supercritical carbon dioxide (SF-CO_2_) extraction allows the solvent to be separated easily by depressurizing the extraction system; however, the process requires high equipment and operating costs due to high pressure application. Subcritical extraction uses a lower pressure (1.0–15 MPa) [22,30] than SFE (41–69 MPa) [31], and subcritical liquefied dimethyl ether (SUBLDME) extraction successfully extracted oil from a variety of materials, including tuna liver, hemp seed, raw macroalgae, rubber seed, green tea leaves, algae, and blue-green microalgae [22,32,33,34,35,36,37]. Fluid carbon dioxide is the extraction state for SF-CO_2_, while liquefied DME is the extraction state for SUBLDME.

DME has a low toxicity, and accordingly, it has been examined as a prospective solvent for use in food processing. It has been approved as a safe solvent for the food industry by the European Food Safety Authority [38], and its residue will not be present in the final extract at room temperature owing to its low boiling point (−24.8 °C) [32]. It has been authorized by the European Food Safety Authority (EFSA) as safe for food preparation of defatted animal protein products (EU Directive 2009/32/EC). Food Standards Australia New Zealand (FSANZ) [38] consent to the use of DME as a processing aid in the production of dairy and non-dairy food ingredients and products [39]. There are many reports on the synthesis of DME from greenhouse gases of carbon dioxide (CO_2_) through methanol [40,41,42,43]. Consequently, utilizing DME to create value added to agricultural waste by extraction of high-value food products is one of the strategies to reduce CO_2_ greenhouse gas emissions. SUBLDME extraction consumes less energy and emits less greenhouse gases than extractions with other solvents, including carbon dioxide [44]. Many reports have revealed the effect of oil extraction from various plants by conventional and unconventional methods on yield and nutraceutical components, as demonstrated in Table 1. However, there is still a lack of data on the application of SUBLDME extraction on oil extraction from rice bran.

Based on color, rice bran can be generally classified into two types, namely bran from white rice and bran from colored rice, such as red rice, black rice, etc. This study was focused on rice bran (*Oryza sativa* L.) from white (Hom Mali 105) and red rice (Riceberry rice). Therefore, the current study aims to compare the nutraceutical composition with respect to γ-oryzanol, phytosterol, and policosanol content as well as the antioxidant properties of crude rice bran oils (CRBO) extracted using green techniques including conventional cold pressing (CPE) and two unconventional green extraction methods of supercritical CO_2_ extraction and subcritical liquified DME extraction, comparing with conventional solvent extraction using hexane.

## 2. Results and Discussion

### 2.1. Effect of Rice Bran Oil Extraction Methods on Yield, and Oil Chemical Qualities

**Figure 1:**
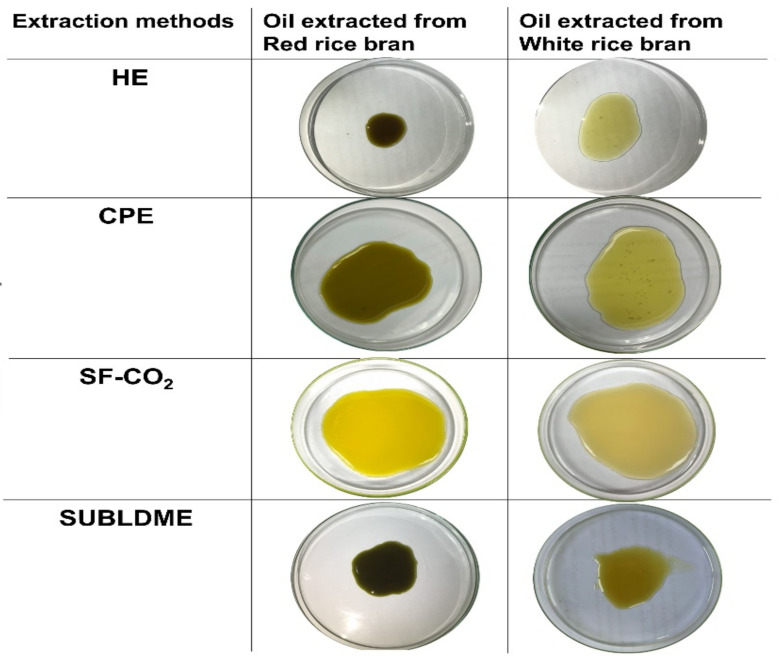
The rice bran oil extracted with hexane extraction (HE), cold-pressed extraction (CPE), supercritical fluid carbon dioxide extraction (SF-CO_2_), and subcritical liquefied DME extraction (SUBLDME).

Two types of bran from white and red rice were extracted using HE, CPE, SF-CO_2_, and SUBLDME methods. The obtained CRBOs are shown in Figure 1, and the extraction yield value, fat contents, and oil chemical qualities in terms of acid value, free fatty acid, and peroxide value are shown in Table 2. The oil yields ranged from 4.2 to 26.0% for the white rice bran and from 5.4 to 20.8% for the red rice bran. The highest oil yield percentage was 26.0% from the solvent extraction of white rice bran, followed by SUBLDME extraction of white rice bran (24.6%), while the lowest yield was 4.2% from the cold-pressed extraction method of white rice bran. For cold-pressed extraction, the oil yields were not significantly different between white and red rice bran (4.2% vs. 5.4%). The oil extraction yields were in the order of HE > SUBLDME > SF-CO_2_ > CPE. For the three green extraction techniques, the CPE gave the lowest oil yield (4.2–5.4%), whereas SUBLDME extraction gave a higher oil yield than SF-CO_2_ extraction. Unlike CPE, SUBLDME and SF-CO_2_ extraction are based on solvent in different extraction phases; therefore, it is possible that the process steps cause release of the oil component from the bran matrix in the different mechanisms. Data also suggest that for all extraction methods except for CPE, white rice bran gave a higher extraction yield than red rice bran. Compared with other extraction methods, CPE was also reported to have the lowest oil yield by Pengkumsri et al. [61] and Mingyai et al. [62]. They reported that the highest yield of rice bran oil was obtained by using HE, followed by SF-CO_2_ and CPE.

The physiochemical properties of CRBO, including acid value (AV), free fatty acid (FFA), and peroxide value (PV) by different methods, were determined and are given in Table 2. The highest AV and FFA were found in the CRBO extracted using SF-CO_2_ methods (48.09 and 49.27 mg KOH/g oil, and 24.17 and 24.77%, respectively). The explanation for why SF-CO_2_ gave the highest AV and FFA compared to other extraction methods was the high pressure used (25 MPa) and the higher solubility of FFA in fluid carbon dioxide, resulting in greater selectivity for the FFA [63]. Similar results have been reported for the supercritical phase of CO_2_ on deacidification studies performed with other vegetable oils [63,64,65]. Arora et al., 2015 [66] recommended that the maximum level of FFA for CRBO was 6–8%. FFA contents of CRBO obtained in this study were slightly higher than the recommendation given by Arora et al. [66]. The main reason for this is the time lag between its production and utilization. If the oil is not extracted immediately after the milling of the rice, it will be hydrolyzed into free fatty acids and glycerol by the action of lipase enzymes. The rate of oil degradation and FFA formation during storage could be very high (about 1–7% per day) under suitable conditions [66,67,68].

Oluremi [69] found that the AV of CRBO was 80.0 mg KOH/g oil. However, according to the CODEX standard, the maximum level of AV obtained by cold-pressed extraction of virgin oils is 4.0 mg KOH/g oil [70]. The AVs for the CRBO found here from all methods were similar with that found by Sawadikiat and Hongsprabhas [71]. They found that the CRBO extracted by solvent contained 21.1 mg KOH/g oil for AV and 10.6% for FFA [71]. The AV and FFA of CRBO extracted by SUBLDME (14.04 and 16.23 mg KOH/g oil as well as 7.06 and 8.16%, respectively) were not significantly different (*p* < 0.05) from the values of CRBO extracted using CPE (14.39 and 16.55 mg KOH/g oil as well as 6.69 and 8.32%, respectively). The FFA value is used as the index of hydrolytic rancidity in the rice bran oil. Lipase, lipoxygenase, and peroxidase are the key factors promoting the hydrolysis of the oil in the bran into glycerol and free fatty acids. Lipoxygenase was generally inactivated after heating at 50−70 °C for 10 min [72]. The AV and FFA were normally related to each other. Compared with the fatty acids in the triacylglycerols, free fatty acids are more susceptible to lipid oxidation, leading to rancidity and production of off-odor, which will also be indicated by PV.

The PVs of red and white CRBOs extracted using HE were the lowest at 0.53 and 0.43 meq/kg, respectively, followed by those extracted using SUBLDME (2.66 and 2.98 meq/kg) (Table 2). Thanonkaew et al. [73] reported that the AV, FFA, and PV of the CRBO extracted from different methods of bran stabilization by CPE were in the range of 11.11–6.98 mg KOH/g, 3.17–5.58%, and 11.72–18.85 meq/kg oil, respectively. The peroxide value is the indicator of lipid oxidation in terms of rancidity. The compounds generated react with low-molecular-weight metals, producing free radicals that are capable of further lipid oxidation [74]. The PV of CRBO must be between 20 and 40 meq/kg [69]. The result here found high AV and FFA compared with the report of Thanonkaew et al. [73]. This is the reason that the bran from their study was stabilized using domestic heating, which is more effective than using the extruder. However, the PV of this study was lower than that of Thanonkaew et al. [73].

The CRBOs from red and white rice bran with the different extraction methods are shown in Figure 1 and the defatted rice bran and the bran before oil extraction by different methods are shown in Figure S1. The color as L and b* value of the oil extracted from red rice bran using SF-CO_2_ was the highest, and the result indicated that the CRBO extracted from red bran using SF-CO_2_ gave the oil color more yellow and brightness than the other methods, which were in accordance with the L, b*, and a* color values in Table 3. The color of CRBO extracted from white rice bran using HE and CPE look similar (Figure 1), consistent with L and a* value of the oil. The color of the oils extracted from red rice bran using SUBLDME and CPE were similar (Figure 1) and also consistent with L and b* values, which were duller yellow compared with other extraction methods (Figure 1 and Table 3). Since red rice bran is rich in pigment and nutraceuticals, the extraction via HE, CPE, and SUBLDME caused these compounds to be released into the CRBO, resulting in a dull color compared with using SF-CO_2_. Riceberry rice was also reported to contain a large amount of pigment as the glycoside form of anthocyanins such as peonidin-3-glucoside (P3G) and cyanidin-3-glucoside (C3G) [75,76,77]. Anthocyanins are regarded as important nutraceuticals mainly due to their antioxidant effect, anti-hyperglycemia, anti-inflammation, anti-hyperlipidemia, and improvement of gut microbiome [77,78,79,80]. Compared with three extraction methods (HE, CPE, and SUBLDME), the principal extraction of SF-CO_2_ was different. It applied high pressure and the carbon dioxide fluid state to extract the oil. 

The CRBO extracted from red rice bran via SUBLDME had the highest viscosity of 225.85 cPs, whereas oil from white bran obtained via HE gave the lowest viscosity (19.73 cPs) (Table 3). There was no significant difference in oil viscosity between red and white bran extracted with SF-CO_2_. The oil viscosity from white rice bran and red rice bran were in the order of SF-CO_2_ > SUBLDME > CPE > HE and SUBLDME > SF-CO_2_ > CPE > HE, respectively. The result suggests that it is possible that HE released more oil content but unsaponifiable matter compared with other extraction methods. The highest viscosity value of oil is partly attributed to unsaponifiable matter, which is numerous, including hydrocarbons, tocopherol, high alcohols, sterols, squalene, ferulic acid esters [81] and to impurity levels such as non-TAG, metal ions, pigment compounds, odors, and gums [82]. The gum of oil extracted from white rice bran by SUBLDME was the highest at 5.44% compared with that by the SF-CO_2_ method (2.27%) (data not shown). In general, the CRBO contains free fatty acid, wax, and unsaponifiable constituents (e.g., γ-oryzanol and tocopherol), phospholipids, glycolipids, and pigments based upon the quality of rice bran [83]. The data agreed with the findings of Jennings and Akoh [84] and Hussain et al. [85], who stated that the viscosity of the CRBO extracted by CPE was 78.60 and 72.3–74.2 cPs, respectively. Gopala [86] also reported that the viscosity of the CRBO extracted by HE was 37 cPs.

### 2.2. Effect of Extraction Methods on Antioxidant Activities of the Crude Rice Bran Oil from White and Red Rice Bran

The total phenolic contents (TPC) and antioxidant properties of the crude RBO had been studied using in vitro assays and the results are shown in Table 4. The TPC, DPPH, and FRAP of the crude oil from white rice bran ranged from 38.71 to 400.39 mg GAE/100 g oil, 5.59 to 16.41 mg Trox E/100 g, and 266.48 to 942.53 mg Trox E/100 g, respectively. The TPC, DPPH, and FRAP of the crude oil from red rice bran ranged from 68.64 to 1880.36 mg GAE/100 g oil, 12.89 to 37.03 mg Trox E/100 g, and 483.24 to 25,304 mg Trox E/100 g, respectively. The results suggested that the crude oil from red rice bran had higher antioxidant capacity than that of white rice bran, and the result here was consistent with the findings on the high viscosity of oil from red rice bran due to unsaponifiable matter containing nutraceuticals such as pigment. The explanation of these phenomena is that the bran from red rice is rich in nutraceuticals and pigments compared to the bran from white rice [61,87,88,89]. The SUBLDME extraction method caused the highest TPC for both oils from the white and colored rice bran (400.39 and 1880.36 mg GAE/100 g oil, respectively). The TPC and FRAP values of the CRBO were in the order of SUBLDME > HE > CPE > SF-CO_2_ (both white and red bran) and SUBLDME > SF-CO_2_ > HE > CPE (white rice bran) as well as SUBLDME > HE > SF-CO_2_ > CPE (red rice bran) for FRAP. The oil extracted from both white and red rice bran by SUBLDME had the highest antioxidant activities in terms of TPC and FRAP among three green extraction technologies. The CPE and HE extraction methods showed the highest DPPH value of oil from white and red rice bran (16.41 and 37.03 mg Trox E/100 g, respectively). However, SUBLDME- and SF-CO_2_-extracted oil showed a low DPPH value compared with CPE and HE. Regardless of the SUBLDME method, the oil extracted by HE had a high value of antioxidants compared with other extraction methods. Pengkumsri et al. [90] also found that oil extracted by HE methods gave high antioxidant activity compared with cold press and SF-CO_2_ extraction.

### 2.3. Effect of Oil Extraction Methods on Nutraceutical Content of the Crude Rice Bran Oil

#### 2.3.1. Effect of Extraction Methods on γ-Oryzanol Contents

**Figure 2:**
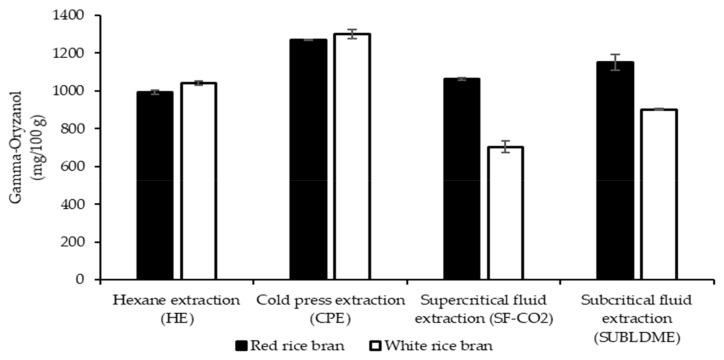
The γ-oryzanol contents of crude rice bran oil extracted using various extraction methods.

Nutraceuticals including γ-oryzanol and phytosterol of the CRBO were analyzed to see the effect of the green extraction methods and the results are shown in Table 5 and Figure 2. Using HPLC analysis, the γ-oryzanol contents of the CRBO extracted from white and red bran by HE, CPE, SF-CO_2_, and SUBLDME were in the range of 741.13–1371.43 mg/100 g. The γ-oryzanol contents of the oil from white and red bran were in the order of CPE > SUBLDME > HE > SF-CO_2_ and CPE > HE > SF-CO_2_ > SUBLDME, respectively. For the three green extraction methods, CPE gave oil with the highest oryzanol, followed by SUBLDME from white rice bran and SF-CO_2_ from red rice bran. Pengkumsri et al. [61] found that the γ-oryzanol contents of CRBO from three different colored rice cultivars (black, brown, and red rice bran) from Thailand using the different extraction methods, including hexane, cold press, hot press, and supercritical fluid extraction, were in range of 175–1849 mg/100 g, and the lowest level of γ-oryzanol was found in the oil extracted by SF-CO_2_ extraction, whereas the highest was found in the oil extracted by hexane extraction [90]. Sawadikiat and Hongsprabhas [71] found that the CRBO obtained using solvent extraction contained a high γ-oryzanol content of 1599 mg/100 g. From this study, the highest γ-oryzanol content of the CRBO was from white rice bran extracted by CPE (1370.43 mg/100 g), while the lowest content was found in oil extracted using SF-CO_2_. The result here is accordance with study of Pengkumsri et al. [61], who found that oryzanol of oil from white rice bran was higher than that of colored rice bran extracted by CPE and SF-CO_2_. Except for the SF-CO_2_ method, HE, CPE, and SUBLDME extraction from white rice bran gave the oil with a high level of γ-oryzanol compared with oil from red rice bran. In contrast to other methods, SF-CO_2_ gave the oil from red rice bran with a higher level of γ-oryzanol than that of white rice bran. Wisetkomolmat et al. [89] studied the proximate analysis of rice bran from non-colored and colored rice and found that non-colored rice bran (three rice cultivars) contained high ash and fat contents (8.22–9.35% and 16.48–18.68%, respectively) compared with the colored rice bran (three rice cultivars) (6.19–7.93% and 12.03–16.48%, respectively). The high ash content found here is the explanation that the oil extracted from white rice bran had higher γ-oryzanol than that of red rice bran [89].

The γ-oryzanol content of the CRBO is based mainly on different extraction methods and the rice cultivars [90]. Pengkumsri et al. [61] also reported that the γ-oryzanol level of CRBO was high following the low color intensity the rice cultivar, in the order of brown, red, and black rice by HE, CPE, hot press, and SF-CO_2_ extraction. The γ-oryzanol content of the oil from white rice bran extracted by SUBLDME was high (1213.64 mg/100 g) compared with SF-CO_2_. The results were comparable to other reports by solvent extraction, cold-pressed extraction, and methanolic subcritical extraction of rice bran oil (1180, 1155, and 1212 mg/100 g, respectively) [91,92,93]. Patel and Naik [94] found γ-oryzanol at 1180 mg/100 g for a mixture of hexane and isopropanol extraction (60 °C) and 5100 mg/100 g for SF-CO_2_ (68.9 MPa at 50 °C). It is possible that the low γ-oryzanol level of the oil found in this study in comparison to Patel and Naik [94] is due to the lower pressure used for SF-CO_2_ (25 vs. 68.9 Mpa) for the same temperature (50 °C) and the different rice cultivar used.

#### 2.3.2. Effect of Extraction Methods on Phytosterol Contents

The phytosterol contents, including campesterol (CAMP), stigmasterol (STGM), β-sitosterol (B-SIT), and sitostanol (SIT), were determined and demonstrated in Table 5. The total phytosterol content of the CRBO from white and red rice bran was in the range of 398.53–1682.21 mg/100 g and 1030.92−1784.17 mg/100 g, respectively. The lowest phytosterol content (398.53 mg/100 g) was found in the crude oil from white rice bran obtained via CPE. The phytosterol content of the CRBO from both white and red rice bran was in the order of SUBLDME > SF-CO_2_ > HE > CPE, and this result suggested that the red rice bran yielded the oil with higher phytosterol content compared with the white rice bran for all extraction methods. Sawadikiat and Hongsprabhas [71] found that the CRBO extracted using solvent extraction contained 1362 mg/100 g of phytosterol, which is consistent with our results. These findings were also consistent with the results of Somseemee et al. [95], who stated that the total phytosterol content of red jasmine rice No. 20-1, 24, and 26 (24.61, 14.65, and 17.32 mg/100 g, respectively) and black jasmine rice No. 12-1 and 13-1 (20.03 and 18.28 mg/100 g, respectively) was higher than white jasmine rice (14.23 mg/100 g). In addition, Mingyai et al. [96] found that RBO from Hom-Nin (a black rice variety) bran had a phytosterol content higher than RBO from Khao Dawk Mali (a white rice variety) bran. For white and red rice bran by all extraction methods, except for red bran extracted using SUBLDME, β-SIT was the major phytosterol found in both the oils from white and red rice bran (145.78–1033.13 mg/100 g and 531.37–955.76 mg/100 g, respectively). Many publications indicated that the β-sitosterol was the predominant sterol in RBO [14,96,97,98,99].

The major phytosterol of crude oil from red rice bran extracted using SUBLDME was CAMP (739.26 mg/100 g). Among the three green extraction methods, SUBLDME gave the oils from both white and red bran the highest total phytosterol (1682.21 and 1784.17 mg/100 g), followed by SF-CO_2_ (1347.33 and 1644.40 mg/100 g, respectively). The maximum phytosterol found in the CRBO extracted using SF-CO_2_ was 1535–1912 mg/100 g [100]. Liu et al. [57] reported that the phytosterol contents of crude RBO by subcritical butane and propane extractions were 2080 and 2010 mg/100 g, respectively. The discrepancies could be attributed to the origins of the rice bran and different types of solvent. Although SUBLDME is wildly used for the extraction of biologically active, lipid, flavoring, or pungent organic compounds in many substances, there are not many studies investigating the effect of the extraction on RBO and phytosterol content. Kamchonemenukool et al. [101] found high phytosterol contents from filter mud discarded from sugar mill extracted using SUBLDME at 20,879 mg/100 g. Guo et al. [45] also reported the phytosterol content of rapeseed cake oil extracted with subcritical 1,1,1,2-tetrafluoroethane (R134a)/butane was 560.19 mg/100 g. Zanqui et al. [102] extracted Brazil nut oil with subcritical n-propane by varying the extraction temperature and pressure, and obtained phytosterol contents ranging from 54.09 to 69.99 mg/100 g. Han et al. [103] also found the phytosterol content of soybean germ oil extracted with subcritical butane at 2772.90 mg/100 g.

#### 2.3.3. Effect of Extraction Methods on Policosanol Contents (PC)

Using GC-MS analysis, the policosanol content of the CRBO from white and red rice bran obtained using different extraction techniques is presented in Table 6. The PC contents of the oil from white and red rice bran ranged from 219.85 to 274.40 mg/100 g and from 205.69 to 254.78 mg/100 g, respectively. The result suggests that oil from white rice bran gave a slightly higher content of PC compared to red rice bran. Like phytosterol content, the PC content of the oil from the white and red rice bran was in the order of SUBLDME > SF-CO_2_ > HE > CPE. The lowest PC content (205.69 and 219.85 mg/100 g for oil from red and white rice bran, respectively) was found in oil extracted using CPE, and among the three green technologies, SUBLDME gave the oil from both white and red bran with the highest total policosanol contents (274.40 and 254.78 mg/100 g, respectively), followed by SF-CO_2_ (255.27 and 254.27 mg/100 g, respectively). From our previous report, the PC content of the 13 commercial rice bran oils was in the range of 18.60–215.20 mg/100 g (data not shown). These findings were consistent with those of Jung et al. [2] and Mingyai et al. [96], who stated that the contents of policosanol in rice bran oil and rice bran were 17.12 and 14.00–26.64 mg/100 g, respectively.

DME exists in a gaseous state at atmospheric conditions (0.1 MPa at 25 °C) and is liquefied at a pressure of more than 0.51 MPa at room temperature, and liquefied DME has a low viscosity, low density, and high solubility in water, or a high degree of miscibility with water, leading to high diffusivity compared with fluid carbon dioxide of SF-CO_2_ method. The density of liquefied DME (1.0 MPa at 60 °C) was about 600 kg/m^3^, whereas that of fluid CO_2_ was around 200–900 kg/m^3^ based on temperature and pressure. The viscosity of liquefied DME is less than 0.15 MPa, while that of fluid CO_2_ is about 0.1–0.3 MPa. Although the mentioned physical properties are similar, the relative permittivity (εr), which is one of the indicators related to the degree of substance polarity of DME, was higher than fluid CO_2_ [101]. The data suggest that the polarity of phytosterol and policosanol are close to liquefied DME compared to γ-oryzanol.

This study focused on investigating effect of green technology on the nutraceutical contents of CRBO. The green technologies CPE, SUBLDME, and SF-CO_2_ had abilities to extract high contents of γ-oryzanol, phytosterol, and policosanol, but to prepare cooking RBO, the crude oil normally passes through the refining processes, leading to the loss of these cholesterol-lowering nutraceuticals. The refining processes of rice bran oil products generate various types of by-products, including gum, rice acid oil, and rice wax. The refining processes are the main factor causing the loss of nutraceuticals into the by-products, especially the loss of PC into rice bran wax and the loss of phytosterol into rice acid oil. Wongwaiwech et al. [104] found a high amount of phytosterol and γ-oryzanol (599.4 and 3901.6 mg/100 g, respectively) in rice acid oil and a high amount of policosanol in rice bran wax (332.8 mg/100 g).

## 3. Materials and Methods

### 3.1. Reagents

All solvents and reagents were of analytical grade or HPLC/GC grade. Hexane (PubChem CID: 8058), ethanol (PubChem CID: 702), and sodium carbonate (PubChem CID: 10340) were purchased from RCI Labscan (Bangkok, Thailand). Dimethyl ether (PubChem CID: 8254) was purchased from Siam Tamiya Co., Ltd. (Bangkok, Thailand). Folin–Ciocalteau reagent (PubChem CID: 516996) was purchased from LOBA Chemie PVT. Ltd. (Maharashtra, India).

Trolox (6-hydroxy-2,5,7,8-tetramethylchroman-2-carboxylic acid) (PubChem CID: 40634), DPPH (1,1-diphenyl-2-picrylhydrazyl) (PubChem CID: 86650676), 2,4,6-tris(2-pyridyl)-s-triazine (TPTZ), 5α-cholestane (PubChem CID: 272895), and phytosterol standards, including campesterol, stigmasterol, β-sitosterol, and sitostanol, and policosanols standards, including tetracosanol (C24) (PubChem CID: 10472), hexacosanol (C26) (PubChem CID: 68171), octacosanol (C28) (PubChem CID: 68406) triacontanol (C30) (PubChem CID: 68972), and dotriacontanol (C-32) (PubChem CID: 96117), and silylation reagent, *N*,*O*-bis(trimethylsilyl)-tri-fluoroacetamide (BSTFA) (PubChem CID: 5366669), with 1% trimethylchlorosilane (TMCS) (PubChem CID: 6397), and pyrogallol (PubChem CID: 1057) were purchased from Sigma-Aldrich (St. Louis, MO, USA). 

DME was purchased from Siam Tamiya Co., Ltd. (Bangkok, Thailand). 2-Propanol (PubChem CID: 3776), potassium hydroxide (KOH) pellets (PubChem CID: 14797), and pyridine (PubChem CID: 1049) were purchased from Merck Millipore (Darmstadt, Germany). Total γ-oryzanol (98.5%) (PubChem CID: 5282164) standard was purchased from Tsuno Rice Fine Chemical Co., Ltd. (Wakayama, Japan).

### 3.2. Materials

Rice bran of Thai non-pigmented rice (white rice bran) (Khao Dawk Mali 105; KDML 105) and Thai red colored rice (red rice bran) (Rice berry; RB) were obtained from Kon mee dee community enterprise in Phitsanulok Province, Thailand. The rice bran obtained by milling for no more than 12 h was dried at 60 °C 24 h to reach a moisture content of 7% in a hot-air oven and then finely milled and sifted through a sieve (60 mesh).

### 3.3. Preparation and Stabilization of Rice Bran Materials

To inhibit enzymes, the obtained rice bran was stabilized with anextrusion machine.]. The extruding process was performed by using a pilot-scale extruder (Naresuan University, Mueang, Phitsanulok, Thailand) with the following conditions: feed rate of 13.25 min/1 kg, speed rate of 20 U, die opening of 10 mm, temperature of 100 °C, followed by cooling at room temperature overnight. The collected rice bran was vacuum-packed in aluminum foil bags and stored at −20 °C until used.

### 3.4. Extraction of the Crude Rice Bran Oils

#### 3.4.1. Cold-Pressing Extraction (CPE)

This operation was carried out three times at a temperature of oil flowing from the press cylinder not exceeding 50 °C, and then fine particles in the expressed oil were separated by filtration. The crude rice bran oil (CRBO) was collected directly from the processing line immediately after extracting operation and stored in an amber vial at −20 °C until used.

#### 3.4.2. Hexane Extraction (HE)

HE was performed by stirring stabilized rice bran in 100% hexane at 50 °C for 1 h. The solvent was removed by vacuum rotary evaporation at 45 °C after filtration through Whatman#4 paper. The obtained CRBO was transferred to an amber bottle and flush with nitrogen gas. The final CRBO was kept at −20 °C until used.

#### 3.4.3. Supercritical Fluid Carbon Dioxide Extraction (SF-CO_2_)

**Figure 3:**
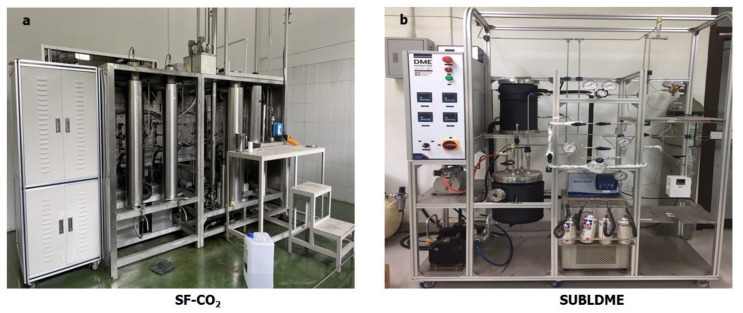
Supercritical fluid carbon dioxide extraction (SF-CO_2_) (**a**); subcritical liquified dimethyl ether extraction (SULDME) (**b**).

SF-CO_2_ extraction was performed without co-solvent application by a pilot-scale supercritical fluid extractor (Guangzhou heaven-sent industry Co., Ltd., Guangzhou, China) with a 5 L extraction kettle, and the schematic diagram of the supercritical CO_2_ extraction system is depicted in Figure 3a. For each extraction, 500 g of samples was placed inside the extractor vessel. Porous stainless steel mesh filters were placed at both ends of the extractor vessel. The extraction was carried out at 25 MPa, 50 °C, at a CO_2_ flow rate of 38–150 L/h for 1 h. To reduce sample variation, 2 batches of extraction were operated. The temperature and pressure of sample collection were 40 °C and 3.8–4 bar (0.38–0.4 MPa), respectively. All extracted CRBOs were stored in amber bottles, purged with nitrogen, and stored at −20 °C for further application.

#### 3.4.4. Subcritical Liquefied Dimethyl Ether Extraction (SUBLDME)

Extractions with DME (Siam Tamiya Co., Ltd., Bangkok, Thailand) were performed with a pilot-scale subcritical dimethyl ether extractor (Naresuan University, Mueang, Phitsanulok, Thailand) as in Figure 3b. Samples (100 g) of the stabilized rice bran were placed into stainless extraction vials and air was exhausted from the system (vacuum pump) to remove air trapped in the system before starting extraction. Nitrogen gas was compressed to increase the pressure of the dimethyl ether storage tank to approximately 1.8 MPa. The pressure in the extraction system was maintained in the range of 1.6–1.8 MPa by the pressure control device (Zhejiang Jiuben Electric Appliance CO., LTD, Zhejiang, China) and the extraction temperature was set to 70 °C for 60 min. The extracted oil was stored in a product tank and the extraction system is equipped with a condenser tank to condense the recycled dimethyl ether gas. To minimized sample variation, three batches were prepared. The obtained CRBOs were stored in stoppered amber glasses, purged with nitrogen, and stored at −20 °C for further analysis.

### 3.5. Chemical Analysis

#### 3.5.1. Acid Value (AV) and Free Fatty Acid Content (FFA) of Oil

The acid values of CRBO samples were determined according to the AOCS Official Method [105]. Briefly, 1 mL of 1% phenolphthalein in ethanol was added to 50 mL of solvent mixture (diethyl ether/ethanol = 1:1) and neutralized to faint pink. Then, 2 g of samples were weighed into an Erlenmeyer flask, to which solvent mixture was added containing phenolphthalein and titrated with 0.1 N KOH. The acid value was calculated and reported as mg KOH in 1 g of sample as shown in Equation (1), and the FFA content was also expressed as the percentage of oleic acid equivalent as shown in Equation (2).

(1) $$\mathrm{Acid}\;\mathrm{value}\;(\mathrm{mg}\;\mathrm{KOH}/g)=\;\frac{\mathrm{KOH}\;\mathrm{solution}\;\left(\mathrm{mL}\right)\times \;\mathrm{Normality}\;\mathrm{of}\;\mathrm{KOH}\;\times 56.1}{\mathrm{Weight}\;\mathrm{of}\;\mathrm{sample}\;\left(g\right)}$$

(2) $$\mathrm{FFA}\;(\%)=\frac{\mathrm{KOH}\;\mathrm{solution}\;\left(\mathrm{mL}\right)\times \;\mathrm{Normality}\;\mathrm{of}\;\mathrm{KOH}\;\times 28.2}{\mathrm{Weight}\;\mathrm{of}\;\mathrm{sample}\;\left(g\right)}$$

#### 3.5.2. Determination of Peroxide Value (PV) of Oil

Titration of CRBO samples was performed with solution (0.01 N) of sodium thiosulphate (Na_2_S_2_O_3_∙5H_2_O). Briefly, 5 g of the sample and 30 mL of the mixture of acetic acid and chloroform (3:2 *v*/*v*) were mixed, and it was shaken gently; then, 0.5 mL of saturated potassium iodide KI solution was added. The mixture was shaken, and then distilled water (30 mL) was added. The mixture was titrated with sodium thiosulphate (0.01 N) until the yellow color of the reactant disappeared. A total of 2 mL of starch solution (1%) was added, which gave a light blue color. The mixture was titrated again until the light blue color discharged [106]. The PV was calculated following Equation (3).

(3) $$\mathrm{Peroxide}\;\mathrm{value}=\frac{V\;\times \;N\;\times 1000}{\mathrm{Weight}\;\mathrm{of}\;\mathrm{sample}}$$

where

V = Difference, in mL, between sample and blank titration;
N = Normality of 0.01 N sodium thiosulphate.

#### 3.5.3. Crude Fat Content of Crude Rice Bran Oil

Fat content was determined by the Soxhlet extraction method, using 250–300 mL of petroleum ether as a solvent. Extractions were carried out using semiautomatic Soxtech Buchi E-816 SOX (Buchi, Flawil, Switzerland) at 155 °C for 2 h. Fat content was calculated as in Equation (4)

(4) $$\%\;\mathrm{Crude}\;\mathrm{fat}=\frac{\left(W2-\;W1\right)\times 100}{\mathrm{Weight}\;\mathrm{of}\;\mathrm{sample}\;\left(g\right)}$$

where

W1 = Weight of empty flask (g);
W2 = Weight of flask and extracted fat (g).

### 3.6. Analysis of Total Phenolic Content (TPC) and Antioxidant Activities

#### 3.6.1. Extraction of CRBO for TPC and Antioxidant Activities Analysis

The CRBO sample (1.5 g) was extracted with methanol 99.9%, and then the extracted solution was used to determine the total phenolic content (TPC) and antioxidant activities of DPPH and FRAP.

#### 3.6.2. Total Phenolic Content (TPC)

The TPC of the extracts was measured using a Folin–Ciocalteau reagent [107]. A quantity of 1 mg of the extract was diluted with 80% ethanol solution. The extract solution was then mixed with 2.5 mL of fresh reagent of Folin-Denis, the final mixture was left in dark conditions for 5 min. An aliquot of 7.5% *w*/*v* of sodium carbonate was added and incubated in dark conditions for 2 h before reading the absorbance at 765 nm (Genesys20, Thermo Scientific, Waltham, MA, USA). The data were expressed as g gallic acid equivalent (GAE) per 100 g of oil.

#### 3.6.3. Total Antioxidant Activity by DPPH Method

DPPH radical scavenging activity was determined using the method developed by Brand-Williams et al. [108]. The working solution of 1,1-diphenyl-2-picrylhydrazyl (DPPH) was freshly prepared in 100% ethanol. The extracted sample (100 µL) was mixed with 100 µL of working DPPH, and the absorbance of the mixture was immediately measured using a spectrophotometer (Genesys20, Thermo Scientific, Waltham, MA, USA) at a wavelength of 517 nm. Trolox was used as a reference antioxidant. All measurements were performed in triplicate. The scavenging effect was derived following Equation (5):

DPPH scavenging effect (%) = [(A_DPPH_ − A_517 nm, sample_)/(A_DPPH_ − A_517 nm, control_)] × 100 (5)

#### 3.6.4. Total Antioxidant Activity by FRAP Method

FRAP assay, originally developed by Benzie and Strain [109], was performed with some modifications. Briefly, a working FRAP reagent was freshly prepared by mixing 25 mL of acetate buffer at pH 3.6, 2.5 mL of 10 mM TPTZ solution, and 2.5 mL of 20 mM ferric chloride hexahydrate (FeCl_3_∙6H_2_O). The working FRAP reagent was mixed with 2850 µL of the extract solution in the test tube and then incubated in dark conditions at room temperature for 15–30 min before reading the absorbance at 595 nm (Genesys20, Thermo Scientific, Waltham, MA, USA). Trolox was used as a reference.

### 3.7. Analysis of Nutraceuticals

#### 3.7.1. Analysis of γ-Oryzanol Contents

γ-Oryzanol was extracted and determined according to a previous report by Wongwaiwech et al. [104] using LC–MS. In brief, the extracted oils were mixed with a solution of acetonitrile, methanol, and isopropanol then injected into the LC–MS. γ-oryzanol was identified by an Agilent Technologies 1100 with a diode array detector (DAD) equipped with a UV detector set at 298 and 325 nm. The analytical column was an Agilent Zorbax Eclipse XDB-C18 column (4.6 mm × 150 mm × 5 µm, Agilent Technologies, Santa Clara, CA, USA). The mobile phase was consisting of acetonitrile, methanol, and isopropanol (25:70:5 *v*/*v*) with a flow rate of 1 mL/min. The temperature of the column was kept at 40 °C. The mass spectrometer was an Agilent Technologies (Santa Clara, CA, USA) LC/MSD SL equipped with an electrospray ion source (ESI) using the following interface parameters: a drying gas flow of 13.01 L/min, nebulizer pressure of 50 psi, drying gas temperatures of 350 °C, a capillary voltage of 4000 V, and recorded on a mass range of *m*/*z* 200–800.

#### 3.7.2. Analysis of Phytosterol Contents

The phytosterol content of CRBO was analyzed using GC–MS according to a previous method [104]. Briefly, oil was extracted with 60% KOH, 95% ethanol, and 10% NaCl under nitrogen gas (N_2_) conditions. The extract was extracted twice with a mixture solution of hexane and ethyl acetate (9:1, *v*/*v*). The upper layer (unsaponifiables) was collected and evaporated to dryness with nitrogen gas (N_2_). The derivatization was performed by *N*,*O*-bis (trimethylsilyl)-trifluoroacetamide. 

The quantification and identification of phytosterol were performed by Agilent Technologies 7683 (Santa Clara, CA, USA) on a fused silica capillary GC column of HP-5MS (30 m × 0.25 mm i.d. × film thickness 0.25 µm) using helium as a carrier gas at 1.5 mL/min. The separation was performed according to the following oven temperature program: the initial temperature was 100 °C, then raised to 300 °C (14 min) at a rate of 10 °C/min. The injection volume was 1.0 μL in split mode (1:50) with the injector temperature set at 270 °C. The MS detection parameters were an interface temperature of 150 °C and a transfer line temperature of 230 °C. The analysis was carried out in triplicate. TMS-phytosterols were identified and quantified by a SIM (single ion monitoring) mode according to their retention times and MS spectra. The *m/z* ratios of the ions used for quantitative analysis were TMS-campesterol (472), TMS-stigmasterol (484), TMS-β-sitosterol (486), and TMS-sitostanol (488) [110].

#### 3.7.3. Analysis of Policosanol Contents (PC)

The PC analysis was performed according to report of Wongwaiwech et al. [104]. The oil sample was extracted with 0.2 M NaOH (10 mL) prepared in methanol aqueous solution (1:3, *v*/*v*). The saponified solution was extracted again with toluene and the unsaponifiable residue was collected and filtered through a 0.45-µm filter. 

GC-MS analyses were performed using a capillary DB-5 ms fused silica capillary column (30 m × 0.25 mm × 0.25 µm, USA) with gas chromatography (Agilent Technologies 6890, Santa Clara, CA, USA) coupled directly to the mass detector with a split ratio of 1:10. Helium was used as the carrier gas, with a constant flow rate of 1 mL/min. The injector and detector temperatures were 350 °C. The oven temperature was programmed from 150 to 320 °C (15 min) at 4 °C. The policosanol compounds were identified by comparing their retention times and molecular target ion, *m/z* at 411 (tetracosanol; C_24_), 439 (hexacosanol; C_26_), 467 (octacosanol; C_28_), 495 (triacontanol; C_30_), and 523 (dotriacontanol; C_32_). The standard and sample chromatogram of oryzanol, phytosterol, and policosanol are shown in Figures S2 and S3, respectively.

## 4. Conclusions

The percentage yield of crude rice bran oil from conventional hexane extraction was not significantly different with from green technology of subcritical liquified dimethyl ether extraction. Cold-pressed extraction is a green technology giving the lowest oil yield but providing the highest γ-oryzanol content. Both phytosterol and policosanol of crude rice bran oil by subcritical liquefied dimethyl ether extraction were significantly higher than other extraction methods. Regardless of the extraction method used, the white rice bran gave a higher level of policosanol, while the red rice bran gave a higher level of phytosterol. The green extraction method of subcritical liquefied dimethyl ether extraction gave the crude rice bran oil high levels of cholesterol-lowering nutraceuticals, including γ-oryzanol, phytosterol, and policosanol, compared with conventional hexane extraction. Consequently, it has the potential to be used as a green method for rice bran oil extraction to maintain the cholesterol-lowering nutraceuticals.

## Figures and Tables

**Table 1:** Extraction yield, nutrients, and nutraceutical composition of different oils and extraction methods.

Plant Matrix	Nutraceutical Composition	Extraction Methods	Yields (%)	Antioxidant Activity	References
Rapeseed cake	- β-carotene content - Phospholipids content - Tocopherols content - Phytosterol content - Fatty acid composition	- Subcritical R134a/butane - SF-CO_2_ - HE	87.76 74.18 74.25		[45]
Almond	- Total phenolics - Phytosterols - Tocopherols - Tocotrienols	- CPE - Hydraulic press extraction - Subcritical butane	44.05 49.71 41.52	TPC (SF-CO_2_ > CPE)	[46]
Sapucaia nuts	- Fatty acid composition - Triacylglycerol composition	- Subcritical propane - SF-CO_2_ (ethanol as co-solvent) - HE	46.22 33.32 49.50		[47]
Tuna liver	- Fatty acid composition - Physicochemical properties	- SUBLDME - SF-CO_2_	17.46 17.51		[32]
Walnut	- Lipid composition - Antioxidant capacity - Tocopherol - Phytosterol - Squalene - Polyphenol	- CPE - Roasting pressing - HE - Subcritical butane - SF-CO_2_	47.04 45.10 58.11 54.84 44.46	DPPH (μmol TE/kg) 247 172 261 272 241	[48]
Radish seed	- Tocopherol content - Fatty acid composition - Phytosterols contents - Acylglycerols contents	- Subcritical propane - Soxhlet extractor - Ultrasound-assisted extraction	14.94 28.40 19.33		[49]
Passion fruit seed	- Total phenolic content values - Antioxidant performance - Anti-bacterial activity	- SF-CO_2_ - Cold maceration - Ultrasonic-assisted extraction	25.90 23.70 23.10	TPC (mgGAE/g) 33.0 284 336	[50]
Wheat germ	- Phytosterols - Tocopherols - Phospholipids	- Subcritical butane - HE - SF-CO_2_	11.69 11.09 10.19		[28]
Wheat germ	- Fatty Acids - Phytosterols - Tocopherols	- Subcritical butane - HE - SF-CO_2_	9.10 9.24 8.78		[23]
Hemp seed	- Chlorophyll - Fatty Acids	- SUBLDME - Petroleum ether - HE	31 23 26		[33]
Pistachio hull	- Phenolic compounds - Gallic acid and its derivative content	- Subcritical water extraction - Ultrasound-assisted solvent extraction	70.9 59.5	TPC (g/kg) 39.3 81.8	[51]
Cashew nut	- Tocopherols - Beta-sitosterol - Lipid composition	- Bligh and Dyer extraction - Compressed extraction method	35.9 33.1	ORAC (μmol/ET.g of TL) 176.3 156.4	[52]
Flaxseed	- Fatty acid - Phytosterol - Carotenoid content	- Subcritical butane - HE - CPE	28.75 27.53 19.56		[53]
Rice bran	- Oryzanol	- SF-CO_2_ - SF-CO_2_ with - Ultrasound	9.94 11.13–12.10	DPPH (%) 71.7 69.3–72.4	[54]
Rice bran	- Oryzanol	- HE - SF-CO_2_	20.5 19.2–20.4		[55]
Rice bran	- Fatty acid composition - Oryzanol	- SF-CO_2_ - Compressed liquefied petroleum gas	12.68 12.07	DPPH (%) 71.67 67.49	[56]
Rice bran	- Fatty acid - Tocopherol and tocotrienol content - Oryzanol - Phytosterol	- Subcritical butane - Subcritical propane - HE	89.11 91.42 67.73		[57]
Rice bran	- γ-oryzanols - Deacidifications of rice bran oil	- SF-CO_2_	15.70		[58]
Rice bran	- Fatty acid - Tocopherol and tocotrienol content - Oryzanol content	- HE - SF-CO_2_	22.07–22.80 13.32–14.5		[59]
Parboiled rice bran	- Tocopherol - Tocotrienol	- SF-CO_2_	10–80		[60]

TPC-total phenolic content; SF-CO_2_-supercritical fluid CO_2_ extraction; CPE-cold-pressed extraction; HE-hexane extraction; SUBLDME-subcritical liquefied dimethyl ether extraction.

**Table 2:** Crude rice bran oil extraction yield, acid value (AV), free fatty acid (FFA), and peroxide value (PV) of crude rice bran oil using different extraction methods.

Extraction Method	Sample	Oil Yield (%)	Acid Value (mg KOH/g Oil)	FFA (%)	Peroxide Value (meq/kg Oil)
HE	Red rice bran	20.8 ± 7.4 ^bc^	13.30 ± 0.23 ^e^	6.69 ± 0.12 ^e^	0.53 ± 0.19 ^f^
	White rice bran	26.0 ± 2.0 ^a^	14.38 ± 0.12 ^d^	7.23 ± 0.06 ^d^	0.43 ± 0.37 ^f^
CPE	Red rice bran	5.4 ± 1.0 ^e^	16.55 ± 0.40 ^c^	8.32 ± 0.20 ^c^	10.77 ± 0.71 ^a^
	White rice bran	4.2 ± 2.1 ^e^	14.39 ± 0.29 ^d^	7.23 ± 0.15 ^d^	9.57 ± 0.34 ^b^
SF-CO_2_	Red rice bran	15.5 ± 3.0 ^d^	48.09 ± 0.62 ^b^	24.17 ± 0.31 ^b^	5.18 ± 0.48 ^c^
	White rice bran	16.2 ± 2.1 ^d^	49.27 ± 0.29 ^a^	24.77 ± 0.14 ^a^	3.94 ± 1.03 ^d^
SUBLDME	Red rice bran	19.6 ± 1.5 ^cd^	14.04 ± 0.35 ^d^	7.06 ± 0.18 ^d^	2.98 ± 0.51 ^de^
	White rice bran	24.6 ± 1.2 ^ab^	16.23 ± 0.37 ^c^	8.16 ± 0.19 ^c^	2.66 ± 0.49 ^e^

HE-hexane extraction; CPE-cold press extraction; SF-CO_2_-supercritical fluid CO_2_ extraction; SUBLDME-subcritical liquefied DME extraction; FFA-free fatty acid value. Values are expressed as mean ± standard deviation. Values bearing different superscripts within the same column for each oil sample are significantly different (*p* < 0.05).

**Table 3:** Color and viscosity value of crude rice bran oil using different extraction methods.

Extraction Methods	Samples	L*	a*	b*	Viscosity (cPs)
HE	Red rice bran	15.53 ± 0.06 ^h^	0.33 ± 0.06 ^h^	−1.00 ± 0.00 ^f^	43.15 ± 0.09 ^f^
	White rice bran	26.00 ± 0.01 ^d^	2.57 ± 0.06 ^c^	8.17 ± 0.06 ^d^	19.73 ± 0.07 ^g^
CPE	Red rice bran	21.84 ± 0.01 ^g^	2.20 ± 0.01 ^e^	−4.38 ± 0.01 ^g^	75.67 ± 1.53 ^e^
	White rice bran	23.20 ± 0.01 ^f^	1.49 ± 0.02 ^g^	−0.94 ± 0.01 ^f^	84.67 ± 1.53 ^d^
SF-CO_2_	Red rice bran	40.87 ± 0.06 ^a^	4.80 ± 0.01 ^a^	30.20 ± 0.10 ^a^	191.00 ± 0.57 ^b^
	White rice bran	37.07 ± 0.12 ^b^	2.27 ± 0.06 ^d^	14.00 ± 0.01 ^c^	186.90 ± 0.71 ^b^
SUBLDME	Red rice bran	24.27 ± 0.06 ^e^	2.10 ± 0.01 ^f^	7.57 ± 0.06 ^e^	225.85 ± 7.00 ^a^
	White rice bran	35.37 ± 0.06 ^c^	4.10 ± 0.01 ^b^	17.83 ± 0.06 ^b^	152.10 ± 1.27 ^c^

HE-hexane extraction; CPE-cold press extraction; SF-CO_2_-supercritical fluid CO_2_ extraction; SUBLDME-subcritical liquefied DME extraction. Values are expressed as mean ± standard deviation. Values bearing different superscripts within the same column for each oil sample are significantly different (*p* < 0.05).

**Table 4:** Total phenolic, DPPH radical scavenging abilities, and ferric reducing antioxidant power of crude rice bran oil extracted by various extraction methods.

Extraction Method	Sample	TPC (mg GAE/100 g Oil)	DPPH (mg Trox E/100 g)	FRAP (mg Trox E/100 g)
HE	Red rice bran	362.05 ± 12.81 ^b^	37.03 ± 1.06 ^a^	1732.13 ± 24.13 ^b^
	White rice bran	190.39 ± 5.85 ^c^	15.92 ± 0.99 ^c^	364.80 ± 5.54 ^g^
CPE	Red rice bran	192.12 ± 2.81 ^c^	26.58 ± 0.37 ^b^	483.24 ± 13.97 ^f^
	White rice bran	66.60 ± 0.40 ^d^	16.41 ± 0.10 ^c^	266.48 ± 2.02 ^g^
SF-CO_2_	Red rice bran	68.64 ± 3.14 ^d^	12.89 ± 0.16 ^e^	1119.71 ± 55.08 ^c^
	White rice bran	38.71 ± 0.52 ^d^	11.40 ± 0.05 ^f^	720.99 ± 24.12 ^e^
SUBLDME	Red rice bran	1880.36 ± 92.80 ^a^	14.63 ± 0.36 ^d^	25,304.69 ± 138.43 ^a^
	White rice bran	400.39 ± 35.71 ^b^	5.59 ± 0.53 ^g^	942.53 ± 48.46 ^d^

HE-hexane extraction; CPE-cold press extraction; SF-CO_2_-supercritical fluid CO_2_ extraction; SUBLDME-subcritical liquefied DME extraction. Values are expressed as mean ± standard deviation. Values bearing different superscripts within the same column for each oil sample are significantly different (*p* < 0.05).

**Table 5:** γ-oryzanol and phytosterol contents (dry basis) of crude rice bran oil extracted using various extraction methods.

Extraction Method	Rice Bran	γ-Oryzanol (mg/100 g)	Phytosterol Content (mg/100 g)				
			CAMP	STGM	B-SIT	SIT	Total
HE	Red rice	1046.10 ± 10.03 ^d^	180.07 ± 2.52 ^c^	280.43 ± 0.83 ^d^	736.40 ± 1.14 ^d^	40.12 ± 0.41 ^g^	1237.02 ± 4.89 ^e^
	White rice	1096.11 ± 10.81 ^c^	74.12 ± 1.07 ^f^	132.04 ± 0.97 ^g^	371.00 ± 1.74 ^g^	46.93 ± 2.96 ^f^	624.09 ± 3.26 ^g^
CPE	Red rice	1338.32 ± 3.50 ^a^	135.64 ± 0.36 ^e^	265.41 ± 1.01 ^e^	597.17 ± 3.87 ^e^	32.69 ± 0.20 ^h^	1030.92 ± 5.45 ^f^
	White rice	1370.43 ± 26.60 ^a^	57.52 ± 0.24 ^g^	25.04 ± 0.03 ^h^	145.78 ± 0.79 ^h^	170.19 ± 1.29 ^b^	398.53 ± 1.88 ^h^
SF-CO_2_	Red rice	1118.49 ± 7.37 ^c^	237.89 ± 0.26 ^b^	391.29 ± 3.82 ^a^	955.76 ± 0.54 ^b^	59.46 ± 0.28 ^e^	1644.40 ± 4.33 ^c^
	White rice	741.13 ± 31.74 ^f^	161.55 ± 0.28 ^d^	319.35 ± 2.68 ^c^	799.44 ± 0.85 ^c^	66.99 ± 0.37 ^d^	1347.33 ± 4.18 ^d^
SUBLDME	Red rice	949.00 ± 44.21 ^e^	739.26 ± 12.18 ^a^	232.32 ± 5.70 ^f^	531.37 ± 0.89 ^f^	281.21 ± 2.17 ^a^	1784.17 ± 7.75 ^a^
	White rice	1213.64 ± 5.25 ^b^	229.85 ± 0.93 ^b^	337.96 ± 5.14 ^b^	1033.13 ± 13.18 ^a^	81.27 ± 0.06 ^c^	1682.21 ± 19.30 ^b^

HE-hexane extraction; CPE-cold press extraction; SF-CO_2_-supercritical fluid CO_2_ extraction; SUBLDME-subcritical liquefied DME extraction; CAMP-campesterol; STGM-stigmasterol; B-SIT-beta-sitosterol; SIT-sitostanol. Values are expressed as mean ± standard deviation. Values bearing different superscripts within the same column for each oil sample are significantly different (*p* < 0.05).

**Table 6:** Policosanol content of crude rice bran oil extracted using various extraction methods.

Extraction Method	Rice Bran	Policosanol Content (mg/100 g)					
		C24	C26	C28	C30	C32	Total
HE	Red rice	60.80 ± 2.26 ^b^	37.98 ± 0.17 ^ef^	47.92 ± 0.10 ^ef^	41.33 ± 0.03 ^e^	42.12 ± 0.11 ^cd^	230.14 ± 2.68 ^d^
	White rice	47.78 ± 1.09 ^d^	43.23 ± 1.06 ^b^	56.45 ± 0.51 ^a^	55.82 ± 0.40 ^a^	43.00 ± 0.36 ^bc^	246.29 ± 0.53 ^c^
CPE	Red rice	41.88 ± 0.48 ^e^	36.94 ± 031 ^f^	46.48 ± 0.10 ^f^	40.51 ± 0.34 ^e^	39.87 ± 0.10 ^d^	205.69 ± 1.34 ^f^
	White rice	43.90 ± 1.40 ^e^	38.32 ± 0.03 ^ef^	48.82 ± 0.71 ^e^	43.80 ± 4.97 ^de^	45.00 ± 1.42 ^b^	219.85 ± 7.11 ^e^
SF-CO_2_	Red rice	58.66 ± 0.18 ^bc^	39.33 ± 0.03 ^de^	50.38 ± 0.44 ^d^	44.90 ± 5.14 ^cde^	54.51 ± 0.23 ^a^	247.78 ± 4.32 ^bc^
	White rice	57.43 ± 1.61 ^c^	41.57 ± 2.03 ^bc^	53.63 ± 0.23 ^b^	50.51 ± 0.01 ^abc^	52.14 ± 0.28 ^a^	255.27 ± 0.10 ^b^
SUBLDME	Red rice	60.38 ± 0.80 ^bc^	40.51 ± 0.02 ^cd^	51.97 ± 1.01 ^c^	48.11 ± 0.46 ^bcd^	53.80 ± 2.35 ^a^	254.78 ± 4.46 ^bc^
	White rice	68.10 ± 1.05 ^a^	45.73 ± 0.25 ^a^	57.20 ± 1.07 ^a^	51.31 ± 0.49 ^ab^	52.06 ± 1.22 ^a^	274.40 ± 1.98 ^a^

HE-hexane extraction; CPE-cold press extraction; SF-CO_2_-supercritical fluid CO_2_ extraction; SUBLDME-subcritical liquefied DME extraction. Values are expressed as mean ± standard deviation. Values bearing different superscripts within the same column for each oil sample are significantly different (*p* < 0.05).

## Data Availability

Data are available from the authors on request.

## Supplementary Materials

The following supporting information can be downloaded at: https://www.mdpi.com/article/10.3390/molecules28062457/s1, Figure S1. The bran from red and white rice and the bran after extraction with different methods.; Figure S2. Chromatograms of standard compound of γ-oryzanol, policosanol and phytosterol.; Figure S3. Chromatograms of γ-oryzanol, policosanol and phytosterol in rice bran oil extracted from white and red rice bran with different methods.
