# Supplementary material for: Bioactives from Crude Rice Bran Oils Extracted Using Green Technology
Source: Molecules. 2023 Mar 7;28(6):2457. doi: 10.3390/molecules28062457 (PMC10057060; doi:10.3390/molecules28062457)
Supplement: Supplementary file 1 [file molecules-28-02457-s001.zip › molecules-2211454-supplementary.pdf]

Bioactives from Crude Rice Bran Oils Extracted Using Green Technology

Donporn Wongwaiwech [1], Sudthida Kamchonemenukool [2], Chi-Tang Ho [3], Shiming Li [4], Nutthaporn Majai [2], Tepsuda Rungrat [5], Kawee Sujipuli [5], Min-Hsiung Pan [6] and Monthana Weerawatanakorn [2,*]

[1] Department of Agro-Industry, Rajamangala University of Technology Lanna Tak, 41/1 Moo 7, Mai Ngam, Mueang, Tak 63000, Thailand
[2] Department of Agro-Industry, Naresuan University, 99 Moo 9, Tha Pho, Mueang, Phitsanulok 65000, Thailand
[3] Department of Food Science, Rutgers University, 65 Dudley Road, New Brunswick, NJ 08901, USA
[4] Department of Food Science, College of Life Sciences, Huanggang Normal University, Huanggang 438000, China
[5] Department of Agricultural Science, Faculty of Agriculture, Natural Resources and Environment, Naresuan University, 99 Moo 9, Tha Pho, Mueang, Phitsanulok 65000, Thailand
[6] Institute of Food Science and Technology, National Taiwan University, No.1, Section 4, Roosevelt Road, Taipei 10617, Taiwan
* Correspondence: monthanac@nu.ac.th; Tel.: +66-0629514194

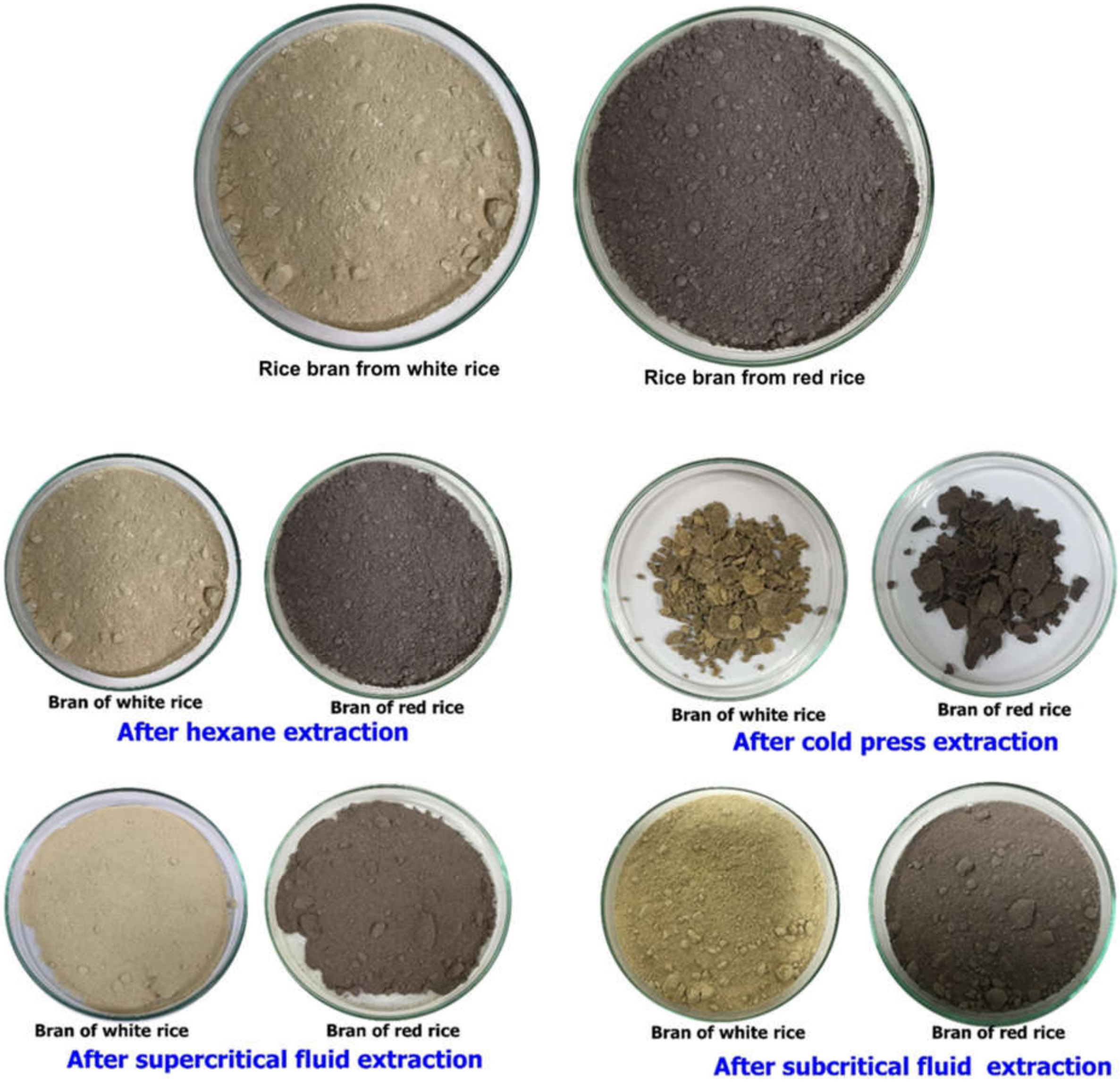

Figure S1. The bran from red and white rice and the bran after extraction with different methods

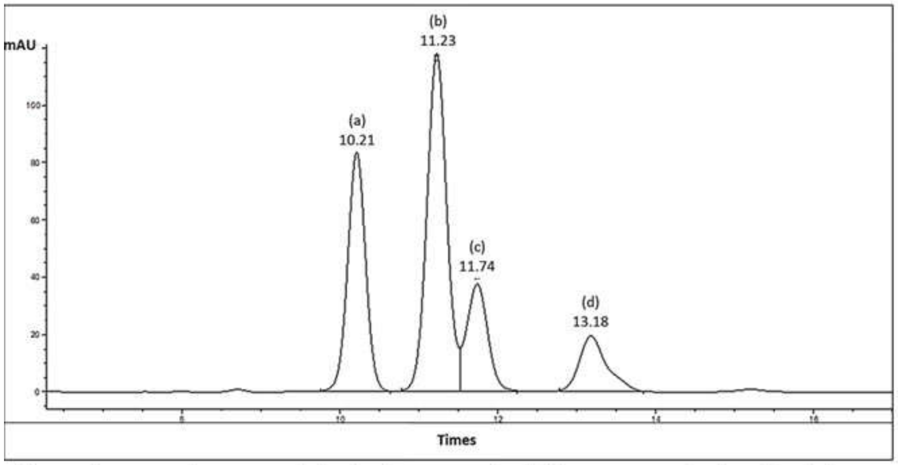

Chromatograms of γ-oryzanol standard compounds at 50 ppm; a. cycloartenyl ferulate, b. 24-methylene cycloartenyl ferulate , c. campesteryl ferulate , d. β-sitosteryl ferulate

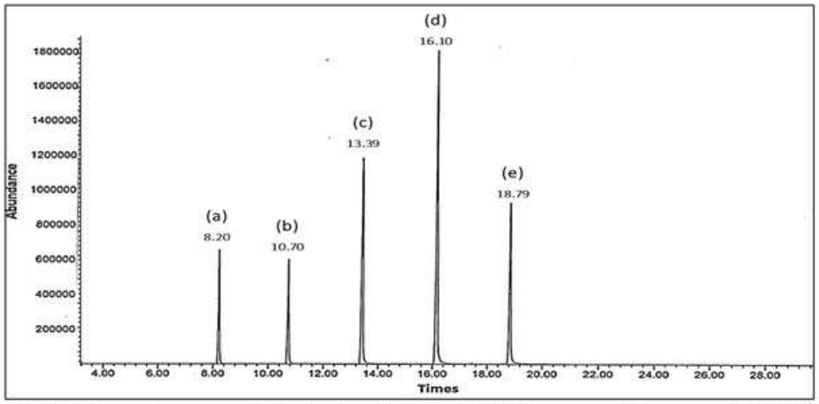

GC-MS chromatograms of standard policosanol at 500 ppm; a. Tetracosanol (C-24), b. Hexacosanol (C-26), c. Octacosanol (C-28), d. Triacosanol (C-30), e. Dotriacontanol (C-32)

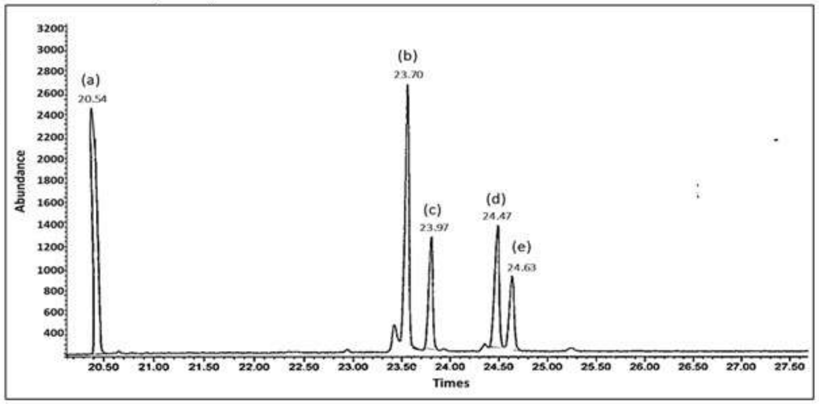

GC-MS chromatograms of standard phytosterol at 100 ppm; a. cholestane as internal standard, b. campesterol, c. stigmasterol, d. b-sitosterol and e. sitostanol

Figure S2. Chromatograms of standard compound of γ-oryzanol, policosanol and phytosterol

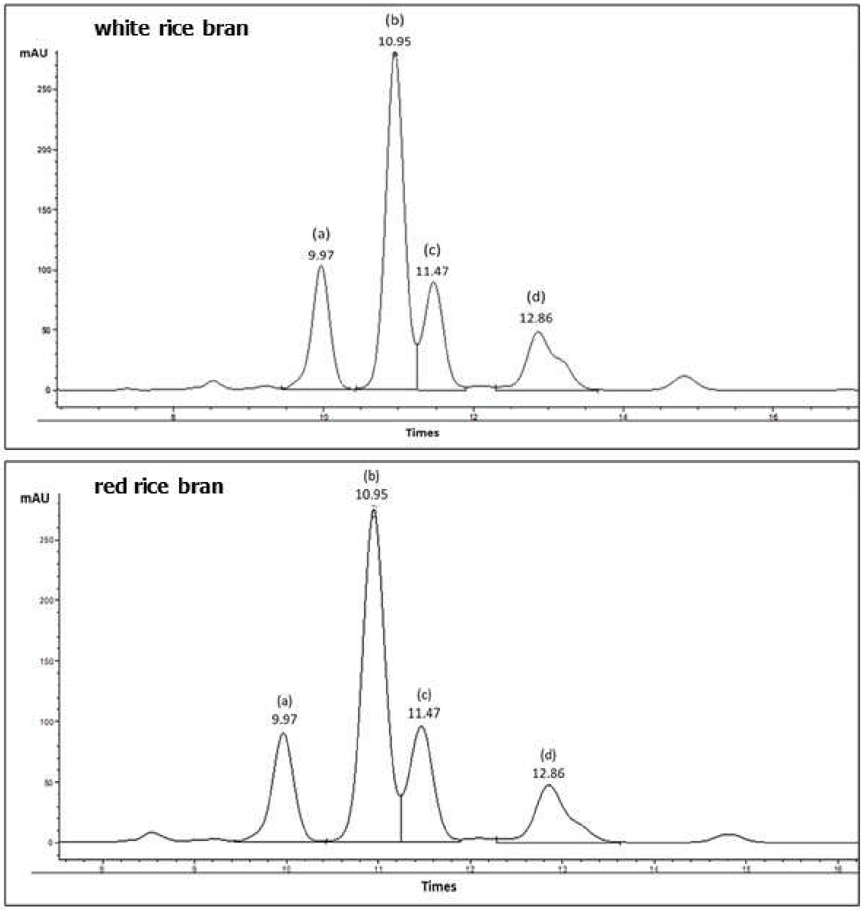

γ-oryzanol chromatogram of RBO from white and red rice bran using cold press extraction method; a. cycloartenyl ferulate, b. 24-methylene cycloartenyl ferulate , c. campesteryl ferulate , d. β-sitosteryl ferulate

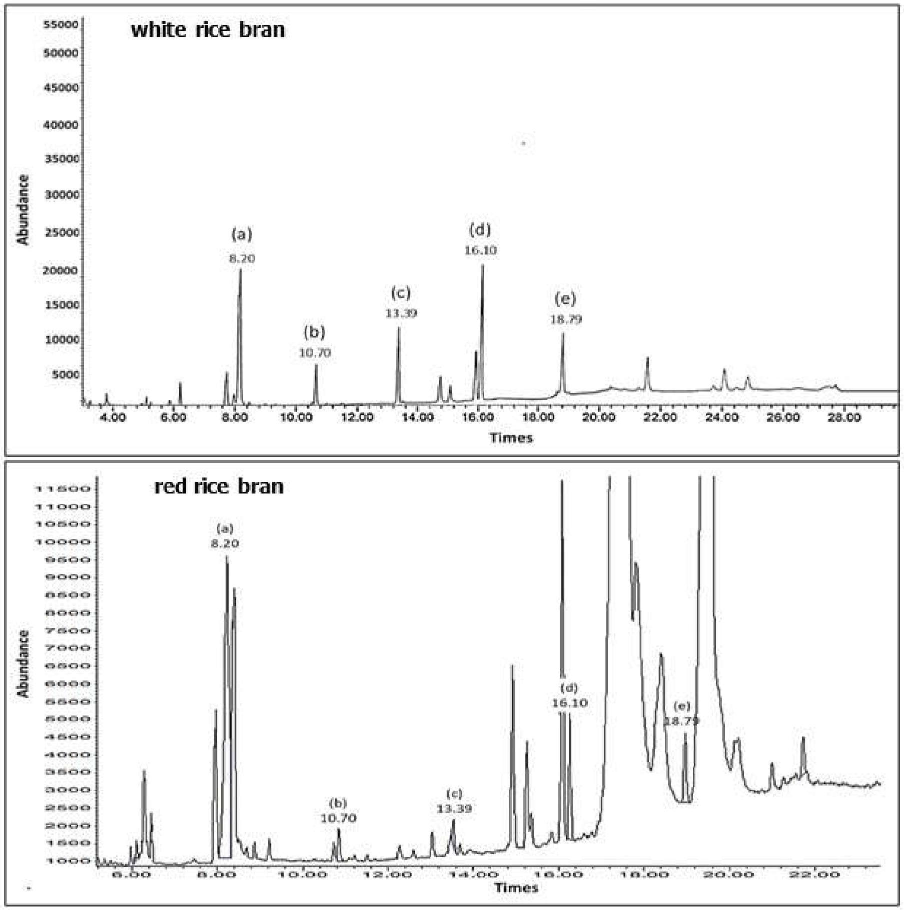

Policosanol chromatograms of RBO from white rice bran using SUBLDME extraction; a. Tetracosanol (C-24), b. Hexacosanol (C-26), c. Octacosanol (C-28), d. Triacosanol (C-30), e. Dotriacontanol (C-32)

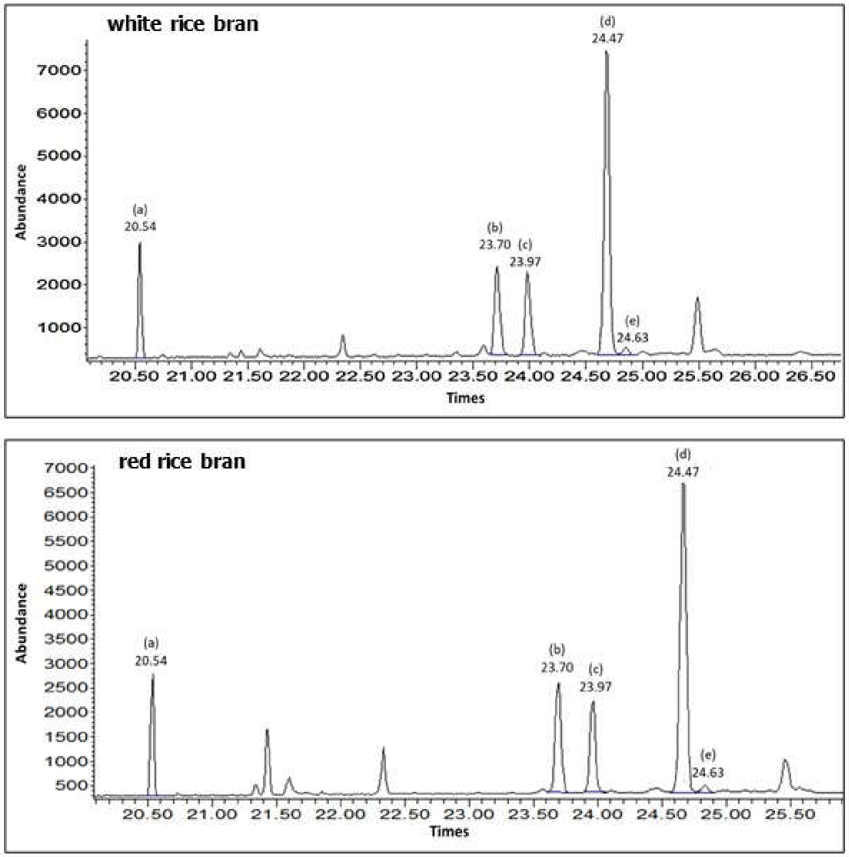

Phytosterol chromatograms of RBO from red rice bran using SUBLDME extraction; a. cholestane as internal standard, b. campesterol, c. stigmasterol, d. b-sitosterol and e. sitostanol

Figure S3. Chromatograms of γ-oryzanol, policosanol and phytosterol in rice bran oil extracted from white and red rice bran with different methods
